# Evaluation of standard and semantically-augmented distance metrics for neurology patients

**DOI:** 10.1186/s12911-020-01217-8

**Published:** 2020-08-26

**Authors:** Daniel B. Hier, Jonathan Kopel, Steven U. Brint, Donald C. Wunsch, Gayla R. Olbricht, Sima Azizi, Blaine Allen

**Affiliations:** 1 Department of Neurology and Rehabilitation, University of Illinois at Chicago, Chicago, IL 60612 USA; 2 Department of Internal Medicine, Texas Tech University Health Sciences Center, Lubbock, TX USA; 3 Department of Electrical and Computer Engineering, Missouri University of Science and Technology, Rolla, MO 65401 USA; 4 Department of Mathematics and Statistics, Missouri University of Science and Technology, Rolla, MO 65401 USA

**Keywords:** Patient distances, Semantic augmentation, Ontologies, Machine learning, Patient clustering, Patient classification, Distance metrics, neurology

## Abstract

**Background:**

Patient distances can be calculated based on signs and symptoms derived from an ontological hierarchy. There is controversy as to whether patient distance metrics that consider the semantic similarity between concepts can outperform standard patient distance metrics that are agnostic to concept similarity. The choice of distance metric can dominate the performance of classification or clustering algorithms. Our objective was to determine if semantically augmented distance metrics would outperform standard metrics on machine learning tasks.

**Methods:**

We converted the neurological findings from 382 published neurology cases into sets of concepts with corresponding machine-readable codes. We calculated patient distances by four different metrics (cosine distance, a semantically augmented cosine distance, Jaccard distance, and a semantically augmented bipartite distance). Semantic augmentation for two of the metrics depended on concept similarities from a hierarchical neuro-ontology. For machine learning algorithms, we used the patient diagnosis as the ground truth *label* and patient findings as machine learning *features*. We assessed classification accuracy for four classifiers and cluster quality for two clustering algorithms for each of the distance metrics.

**Results:**

Inter-patient distances were smaller when the distance metric was semantically augmented. Classification accuracy and cluster quality were not significantly different by distance metric.

**Conclusion:**

Although semantic augmentation reduced inter-patient distances, we did not find improved classification accuracy or improved cluster quality with semantically augmented patient distance metrics when applied to a dataset of neurology patients. Further work is needed to assess the utility of semantically augmented patient distances.

## Background and related work

Patients present with signs (what the physician finds on examination) and symptoms (patient complaints). We group *signs and symptoms* under the more general term *findings* [1]. Distance metrics play an important role in advancing precision medicine, machine learning, and patient phenotyping [2–12]. Patient distances can be calculated based on findings that have been converted to machine codes based on concepts from a hierarchical ontology.

$$ \boldsymbol{signs}+\boldsymbol{symptoms}=\boldsymbol{findings}\rightarrow \boldsymbol{concepts}\rightarrow \boldsymbol{machine}\ \boldsymbol{codes}. $$

In this study, we examine whether the semantic augmentation of distance metrics with concept similarities improves the classification and clustering of neurology patients.

### Distance metrics

A variety of similarity and distance metrics are available. These have been used to calculate distances between patients [13–16], documents [17–19], and phenotypes [4, 5, 9, 10, 12]. If similarity and distance metrics are normalized to a scale of 0.0 to 1.0, the distance between A and B is the complement of the similarity.

1 $$ \mathrm{distance}\ \left(\mathrm{A},\mathrm{B}\right)=1-\mathrm{similarity}\ \left(\mathrm{A},\mathrm{B}\right). $$

The distance between two patients is different than the distance between two medical concepts. Patients are complex and can be represented as a collection of many concepts. Inter-patient distances are many-to-many comparisons; inter-concept distances are one-to-one comparisons. Metrics that work for concept distances are generally different from metrics to calculate distances between patients. Melton et al. [16] comment that “semantic distance measures the relative closeness between two concepts …. Inter-patient distance compares the relative closeness between two cases (sets of patient data).”

The implementation of distance metrics for neurological patients based on findings is challenging. First, neurological findings are recorded as unstructured free text. Second, examiners use a variety of equivalent terms to represent the same meaning: hyperreflexia is equivalent to increased reflexes; Babinski sign is equivalent to extensor plantar response; and so on. Third, the number of findings may vary from patient to patient. Fourth, converting unstructured text into machine-readable codes is difficult [20, 21].

The SNOMED CT ontology and the UMLS Metathesaurus allow the consolidation of multiple synonymous terms under the same concept [22, 23]. Both terminologies assign unique machine-readable codes to a concept. We have identified 1204 core concepts from the UMLS Metathesaurus as a neuro-ontology for capturing findings of the neurological examination [24]. This curated neuro-ontology has three characteristics that make it well-suited for patient distance calculations: 1) it is monohierarchic, 2) the neurologic similarity of concepts has organized its hierarchy, and 3) it contains neurologic concepts absent from SNOMED CT [24].

When findings are converted to concepts and represented as machine-readable codes, patients can be instantiated mathematically as a set (an unordered collection of findings) or as a vector (ordered array of elements of fixed length). If a patient is represented as a set, each finding is added to the set as a unique element. The cardinality of the set (number of set elements) is equal to the number of findings. If a patient is represented as a vector, each finding is represented as an element of the vector. The number of elements is equal to the number of potential findings. A variety of distance metrics can be used with vectors, including Manhattan, Euclidean, cosine, Pearson correlation, Hamming, Minkowski, and others [25]. Commonly used distance metrics in patient similarity studies are Jaccard, Mahalanobis, Euclidean, and cosine [15, 26]. Haase et al. [27] have suggested a bipartite matching algorithm for set similarity (eq. 2) where |A| is the number of elements in set A and sim(a, b) is the similarity between a concept *a* from set *A* and *b* is a concept from set B.

2 $$ Sim\ \left(A,B\right)=\frac{1}{\mid A\mid}\ast \sum \limits_{a\in A}{\mathit{\max}}_{b\in B}\left( sim\left(a,b\right)\right). $$

Bipartite similarity metrics resembling eq. 2 have been used to calculate patient distances [16].

Hierarchical ontologies such as SNOMED CT and the UMLS Metathesaurus allow the calculation of distances between concepts [28–36]. Concept distances derived from hierarchal ontologies show modest correlations with the distance judgments of human experts [35, 37, 38]. The distance metrics for both sets and vectors can be augmented by considering the similarity between concepts [13, 14, 19]. Melton et al. [16] compared computed patient distances with an expert opinion on patient distance based on chart review. They did not find that semantic augmentation of the distance metric enhanced correlation with expert opinion and that correlation between experts and computed patient distances was low regardless of semantic augmentation. Mabotuwana et al. [19] examined document similarity using a cosine distance metric after converting document concepts to a binarized vector. In a classification task that involved determining whether a radiological report was a head CT scan or an abdomen CT scan, they found the accuracy of a k-nearest neighbor classifier increased from 86.7 to 93.1% with semantic augmentation of the document vector based on the SNOMED CT concept hierarchy. Mabotuwana et al. found that semantic augmentation of inter-document distances increased the separation between the centroid of the head CT scan reports and the centroid of the abdomen CT reports. Jia et al. [14] examined the ability of patient distances generated by ICD-10 diagnoses to predict hospital length of stay. Although they explored a variety of distance metrics, including cosine, Jaccard, and bipartite matching, they came to no definite conclusion as to whether semantic augmentation (based on a concept hierarchy) improved classification accuracy. In the Human Phenotype Ontology (HPO), Kohler et al. [12] have implemented a semantically augmented distance metric to assist in matching unknown patients to archetypical patients in the Online Mendelian Inheritance in Man (OMIM) database. Girardi et al. [13] calculated distances between patients with diseases of the gall bladder, thyroid, or appendix and hernias based on ICD-10 diagnosis codes. They found that a semantically augmented patient distance metric outperformed a Jaccard distance on a clustering task and that a semantically augmented patient distance increased the distance between within-diagnosis centroids and between diagnosis centroids.

### Machine learning

Machine learning is increasingly used in the analysis of patient data. Machine learning is divided into supervised and unsupervised learning [39]. The prototypical tasks for supervised learning are classification and regression [40]. Although there are many machine learning classifiers, some commonly used classifiers include naïve Bayes, logistic regression, k-nearest neighbor, and random forest [40]. Naïve Bayes utilizes probabilities derived from predictor variables to select class membership. Logistic regression is a statistical method that fits parameters to a logistic equation to predict class membership. k-nearest neighbor classifiers utilize distances between cases to predict class membership. Random forest classifiers use an ensemble of decision trees to predict class membership. The most common use of unsupervised learning algorithms is for the clustering of cases into homogeneous groups. Although many clustering algorithms are available, two of the most commonly used clustering algorithms are k-means clustering and agglomerative clustering [41]. Both of these algorithms utilize inter-case distances to form homogeneous clusters of cases. Indices of machine learning classification quality include precision, recall, F1, and accuracy [42]. Indices of machine learning clustering quality include homogeneity, completeness, Rand index, V-score, silhouette score [43–45]. Distance metrics are frequently used to generate patient distance matrices that drive the clustering or classification of patients. Since the performance of machine learning clustering and classification algorithms can be assessed objectively, we have hypothesized that the semantic augmentation of distance metrics with inter-concept distances would improve the performance of these algorithms.

To test this hypothesis, we created four test groups of patients abstracted from textbooks. We investigated four classifiers (naïve Bayes, logistic regression, random forests, and k-nearest neighbor) and two clustering algorithms (agglomerative and k-means) across four distance metrics. We tested whether semantic augmentation of the distance metrics improved clustering or classification quality.

## Methods

### Case abstraction

We created a dataset of 382 neurological patients selected from a convenience sample [46] of 1028 published teaching cases [47–58]. We abstracted 2616 findings from the case studies (mean 6.7 ± 3.4 findings per patient). Findings were transcribed verbatim from source materials. An abstractor manually selected one of the 1204 available terms in the neuro-ontology that best represented the finding and added the UMLS CUI code [24]. Table 1 illustrates the case abstraction method for a patient with Parkinson disease*.*

**Table 1:** Illustration of case abstraction method. The first column is findings from a case of Parkinson disease in **Neuroanatomy through Clinical Cases** [47] and is reproduced with the permission of the author. The second column is the abstractor’s interpretation of the finding, and the third column is the UMLS CUI [24]

Original Finding	Interpretation	CUI
“micrographia”	*micrographia*	**C0240341**
“mask-like decreased facial expression”	*mask-like facies*	**C0424448**
“asymmetrical bradykinesia”	*bradykinesia*	**C0233565**
“cogwheel rigidity”	*cogwheel rigidity*	**C0151564**
“en bloc turning”	*difficulty turning body*	**C0555095**
“Exhibited retropulsion of two steps when pulled gently backward”	*retropulsion*	**C0277845**
“no extinction of the glabellar reflex (Myerson sign)”	*Myerson sign*	**C4293666**
“4 Hz tremor of the head and all extremities, worse at rest”	*resting tremor*	**C0234379**
“Slow, stiff gait with stooped posture, short steps, decreased arm swing”	*decreased arm swing*	**C2938985**
	*stooped posture*	**C4476759**
	*slow gait*	**C1851908**
	*marche a petit pas*	**C0427169**

### Distance metrics

We implemented four inter-patient distance metrics in Python [59]. The *Jaccard distance* is the complement of the Jaccard similarity [60]. If A and B are the sets of findings from patient A and patient B, the Jaccard_dist_ (A, B) is shown by eq. (3), and J_sim_ is the Jaccard similarity.

3 $$ {\mathrm{J}\mathrm{accard}}_{dist}\left(\mathrm{A},\mathrm{B}\right)=1-{\mathrm{J}}_{\mathrm{sim}}\left(\mathrm{A},\mathrm{B}\right)=1-\frac{\mathrm{A}\cap \mathrm{B}}{\mathrm{A}\cup \mathrm{B}}. $$

The *augmented bipartite distance* is based on the metric of Melton et al. [16] after augmenting it with the inter-concept distance proposed by Wu and Palmer [29]. If patients A and B are represented as a set of findings such that a *ϵ* A and b *ϵ* B, the augmented bipartite distance is shown by eq. (4) and is supported by eqs. (5), (6), and (7).

4 $$ \mathrm{agumented}\ \mathrm{bipartite}\ \mathrm{distance}\ \left(\mathrm{A},\mathrm{B}\right)=\frac{\mathrm{D}\ \left(\mathrm{A},\mathrm{B}\right)+\mathrm{D}\ \left(\mathrm{B},\mathrm{A}\right)}{2}. $$

5 $$ D\left(A,B\right)=\frac{1}{\left|A\right|}\ast \sum \limits_{a\epsilon A}{\mathit{\min}}_{b\epsilon B}\ \mathrm{dist}\ \left(\mathrm{a},\mathrm{b}\right). $$

6 $$ D\left(B,A\right)=\frac{1}{\mid B\mid}\ast \sum \limits_{b\epsilon A}{\mathit{\min}}_{a\epsilon B}\  dist\ \left(a,b\right). $$

7 $$ dist\ \left(a,b\right)=1-\frac{2\ast depth(LCS)}{depth\ (a)+ depth(b)}. $$

For eq. (7), we used the hierarchical structure of the neuro-ontology and the method of Wu and Palmer [29] to calculate the *dist (a, b)* as the semantic distance between concept *a* and concept *b*. *LCS* is the lowest common subsumer in the hierarchical ontology for concepts a and b; *depth(a)* is the number of levels from the root concept to concept a; *depth (b)* is the number of levels from the root concept to concept *b*, and *depth (LCS)* is the number of levels from the root concept to the *LCS*. Based on eq. (7), the dist (a, b) for each inter-concept distance was stored as a *nxn* lookup table where the number of possible concepts was *n* = 1204. Values from this lookup table were used in eqs. (5) and (6) to iteratively find the minimum inter-concept distance for each concept from patient A compared to the concepts in patient B. *Cosine distances* between patients (1 – cosine similarity) were calculated by standard methods (eq. 8). If patient A and patient B are represented as vectors of findings from a_1_ to a_n_ and from b_1_ to b_n_, the vector is binarized, so that a_i_ or b_i_ is 1 if the finding is present and 0 if the finding is absent. Patient vectors were represented as a one-dimensional array of length n = 1204, where n is the potential number of findings.

8 $$ cosine\ distance\left(A,B\right)=1-\frac{\sum \left({a}_i\ast {b}_i\right)}{\left(\sqrt{\sum {\mathrm{a}}_{\mathrm{i}}^2}\right)\ast \Big(\sqrt{\sum {\mathrm{b}}_{\mathrm{i}}^2\Big)}}. $$

We calculated an *augmented cosine distance* between patients according to the method of Mabotuwana et al. [19] Patients were represented as one-dimensional arrays as in the cosine distance above. We used the hierarchical structure of the neuro-ontology [24] to find an ordered list of ancestors for each concept. For each of the 1204 concepts in the neuro-ontology, we created a semantically augmented vector. The formula for augmentation was 1/(1 + n) where *n* = 0 for the index concept, *n* = 1 for the parent concepts, *n* = 2 for the grandparent concepts, etc. Descendent concepts (children) in the neuro-ontology were not augmented. Ancestor hierarchy was determined by the neuro-ontology, which is mono-hierarchical [24]. Augmentation vectors were stored in an n*xn* lookup table (*n* = 1204). Semantically augmented patient vectors were created for each patient by traversing a list of concepts for each patient and adding the augmented concept vector to the patient vector to obtain a summary patient vector. After semantic augmentation of the vectors, inter-patient distances were calculated by eq. 8.

For all metrics, distances were positive, symmetric, and normalized between 0.0 and 1.0. Distances for each distance metric were stored in a square *nxn* matrix (*n* = 382 patients) before input to classification or clustering algorithms.

### Test groups

We divided the dataset of 382 patients into four test groups by diagnosis (Table 2). Each test group consisted of patients with eight related diagnoses. Each diagnosis occurred at least four times (mean 11.9 ± 5.9) in the test group. Test groups were composed of competing diagnoses for a common presenting neurological complaint (*a patient with weakness, a patient with abnormal movements, a patient with altered mental status, and a patient with cranial neuropathy*). Diagnoses were selected to emulate the differential diagnosis a neurologist might consider when evaluating a patient complaint.

**Table 2:** Four test groups and 32 diagnoses used in clustering and classification analyses. The first column is an abbreviation used in Tables and Figures. Typical findings are listed illustratively for non-neurologists and are not meant to be a definitive reference on each condition

	Test Group	Typical Findings	N
	Patient with weakness	Group 1	148
**GBS**	Guillain Barré syndrome*	weakness, areflexia, sensory loss, paresthesias	20
**MYL**	myelopathy	weakness, sensory level, urinary retention, hyperreflexia	29
**CE**	cauda equina	leg weakness, urinary retention, sensory loss	6
**ALS**	amyotrophic lateral sclerosis	weakness, hyperreflexia, fasciculations	21
**MS**	multiple sclerosis	weakness, sensory changes, hyperreflexia, diplopia	19
**MYO**	myopathy	proximal muscle weakness	15
**MG**	myasthenia gravis	weakness, diplopia, ptosis	18
**PN**	polyneuropathy	weakness, sensory loss, hyporeflexia	20
	**Patient with abnormal movements**	**Group 2**	**75**
**HD**	Huntington disease*	chorea, personality change	16
**PAR**	Parkinson disease*	tremor, bradykinesia, rigidity	19
**PSP**	progressive supranuclear palsy	bradykinesia, rigidity, gaze palsies	8
**SND**	striatonigral degeneration	bradykinesia, rigidity	8
**ET**	essential tremor	tremor	7
**HB**	hemiballismus	hemiballismus	4
**DYS**	dystonia	dystonia	9
**WIL**	Wilson disease*	tremor, ataxia, dystonia, bradykinesia, personality change	4
	**Patient with altered mental status**	**Group 3**	**102**
**LBD**	Lewy body dementia	dementia, bradykinesia, hallucinations	6
**B12**	B_12_ deficiency	paresthesias, confusion, weakness, sensory loss	9
**NPH**	normal pressure hydrocephalus	urinary incontinence, dementia, gait apraxia	14
**AW**	acute Wernicke encephalopathy*	confusion, diplopia, ataxia, disorientation	19
**CJD**	Creutzfeldt-Jakob disease*	myoclonus, personality change, memory loss, disorientation	12
**ALZ**	Alzheimer disease*	amnesia, dementia	16
**FTD**	frontotemporal dementia	aphasia, dementia, executive dysfunction	14
**SDH**	subdural hematoma	headache, lethargy, weakness, confusion	12
	**Patient with cranial neuropathy**	**Group 4**	**67**
**BPV**	benign positional vertigo	vertigo	9
**MNR**	Meniere disease*	vertigo, dizziness, hearing loss	7
**RH**	Ramsay Hunt syndrome*	facial weakness, hearing loss	6
**BEL**	Bell palsy*	facial weakness	10
**THD**	third nerve palsy	diplopia, ptosis	8
**AN**	acoustic neuroma	tinnitus, hearing loss, nystagmus	11
**ON**	optic neuritis	blurred vision, papilledema	6
**TN**	trigeminal neuralgia	face pain	10

*The non-possessive form of eponymous diseases has been used uniformly [61]

### Classification and clustering

For the classification tasks, we assessed the ability to assign correctly *diagnoses* based on *findings*. The ground truth labels were the diagnoses from the abstracted patient histories, and the features were the abstracted findings. Naïve Bayes, logistic regression, random forest, and k-nearest neighbor classifiers were compared. We used the Orange 3.25 default hyperparameters for naïve Bayes. For logistic regression, we set regularization = L2, and for random forest, we set the number of trees = 10. For the k-nearest neighbor classifier, we used uniform distance weighting and k = 5 after the empirical evaluation of all k values between 2 and 15. We used classification accuracy and a balanced F1 score to assess classification performance based on 10-fold cross-validation [42]. In a separate analysis, we found mean F1 scores and mean accuracy scores did not differ statistically (df = 1, *p* > .05) between the 10-fold cross-validation method and the random sampling validation method.

For both the agglomerative clustering algorithm (Ward linkage) [62] and the k-means clustering algorithm, we chose a hyperparameter of *number of clusters = 8* based on the known number of diagnoses in the test groups (Table 2). We used the silhouette score, homogeneity score, completeness score, V-score, adjusted Rand index, and mutual information index to assess cluster quality [42–45, 59].

### Statistical methods

We used SPSS 26 (IBM Corporation) for analysis of variance, line plots, and box plots. We used Orange 3.25.0 for the k-nearest neighbor, logistic regression, naïve Bayes, and random forest classifications. We used scikit-learn 0.23.1 for agglomerative clustering and k-means clustering [59]. All performance measures for clustering and classification were normalized to a 0 to 100 scale.

## Results

**Fig. 1:**
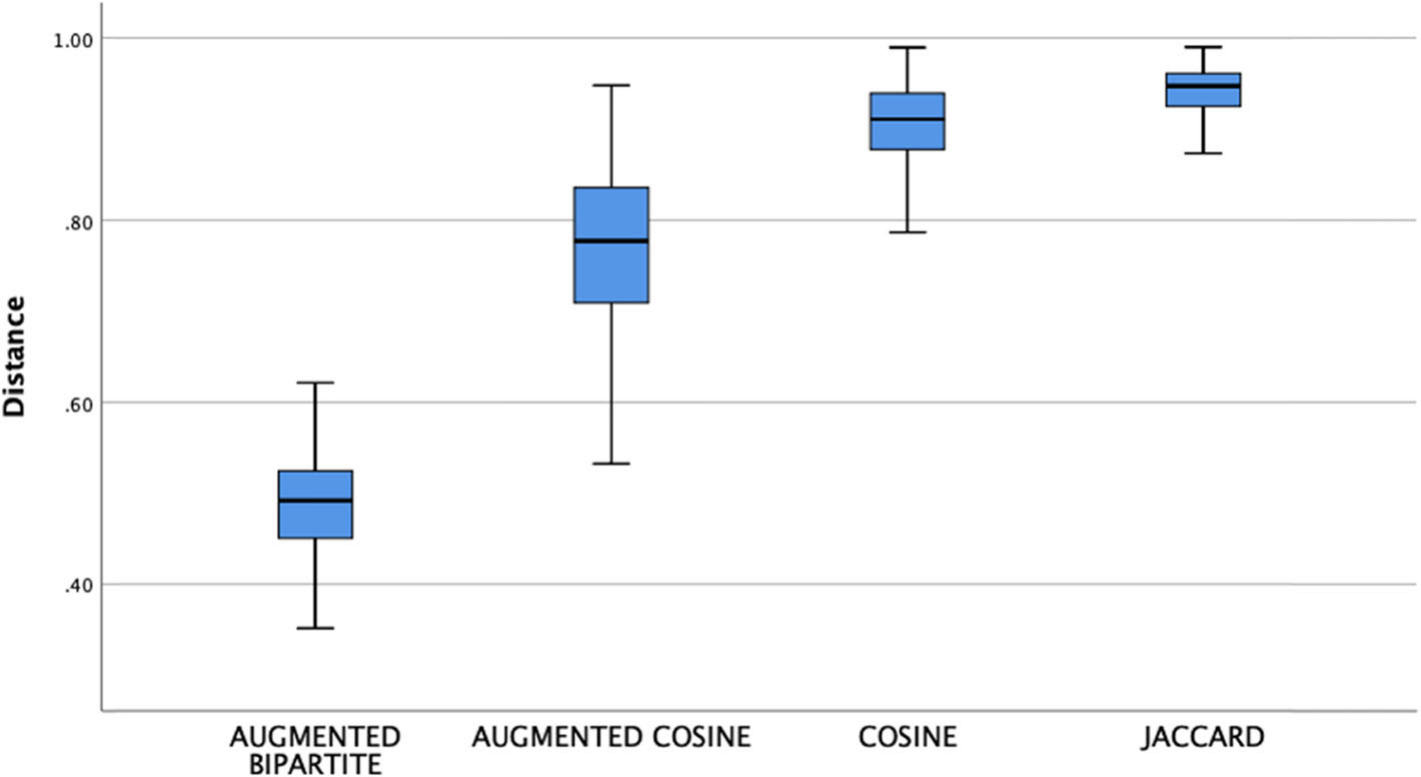
Box-plots inter-patient distances by metric. Means differ by distance metric, (one-way ANOVA, df = 3, F = 5820, *p* < .001). All of the means differed by Bonferroni post hoc test (*p* < .05) with the Jaccard distance the largest and the augmented bipartite the smallest

We examined inter-patient distances for 382 patients divided into 4 test groups of eight diagnoses (Table 2). Inter-patient means differed by distance metric (Fig. 1, one-way ANOVA, df = 3, F = 5820, *p* < .001). Post hoc means testing (Bonferroni *p* < .05) showed all means differed (p < .05) with the augmented bipartite distance metric having the lowest inter-patient mean distance and the Jaccard distance metric having the highest mean inter-patent distance.

**Fig. 2:**
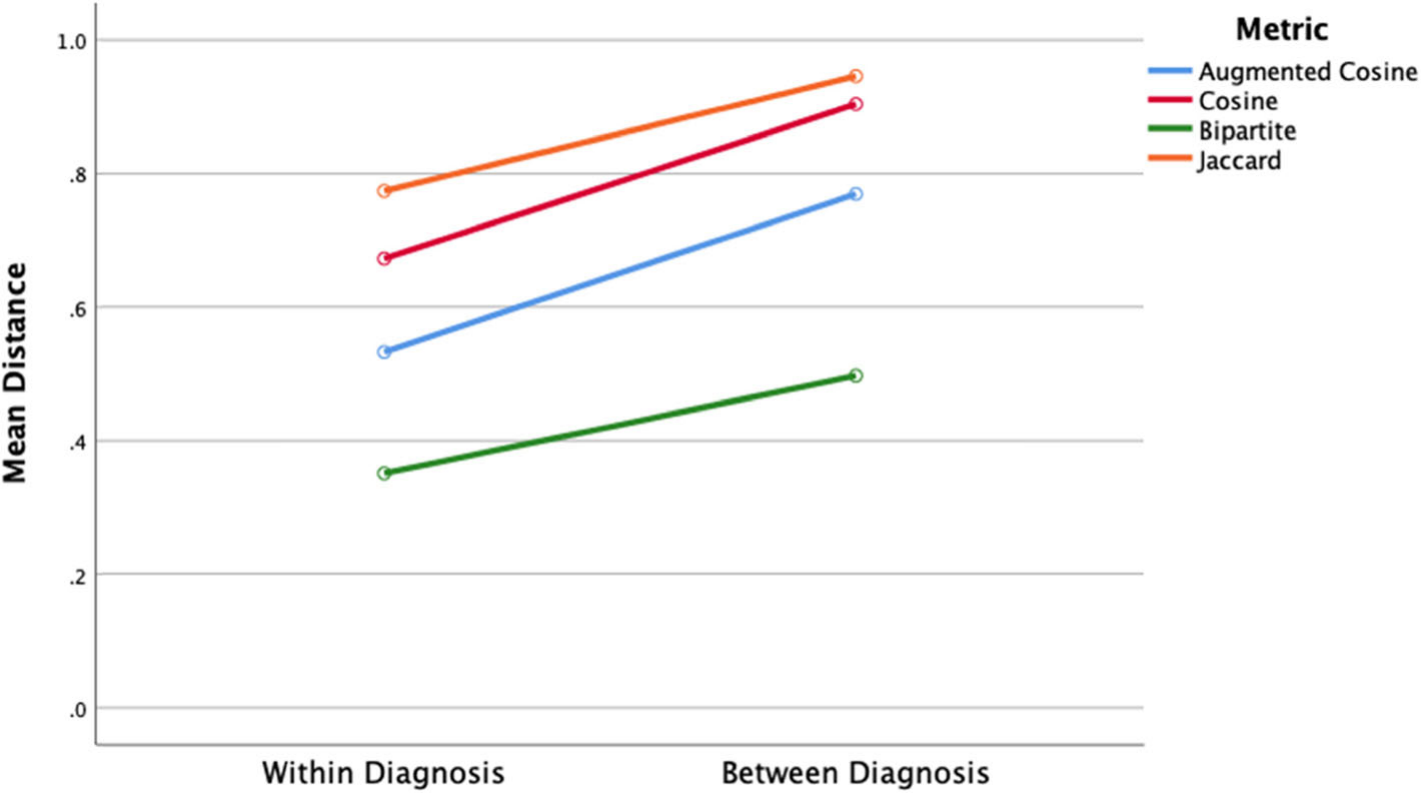
Mean within-diagnosis distance compared to mean between-diagnosis. The within-diagnosis means offer information on patient-to-patient variability within a diagnosis; between-diagnosis means offers information on the degree of separation between patients with one diagnosis from patients of another diagnosis. Mean inter-patient distances were highest for cosine and Jaccard metrics, lowest for augmented bipartite and augmented cosine metrics (post hoc Bonferroni test, p < .05). Within-diagnosis mean distances are lower than between-diagnosis mean distances for all metrics (post hoc Bonferroni test, p < .05)

The mean within-diagnosis patient distance was less than mean between-diagnosis patient distance for all the four-distance metrics (Fig. 2, two-way ANOVA, means differ by group, df = 1, F = 3050, *p* < .001 and means differ by distance metric, df = 3, F = 2936, p < .001). All pairwise mean comparisons by the group and by distance metric were significant (post hoc Bonferroni test, *p* < .05).

**Fig. 3:**
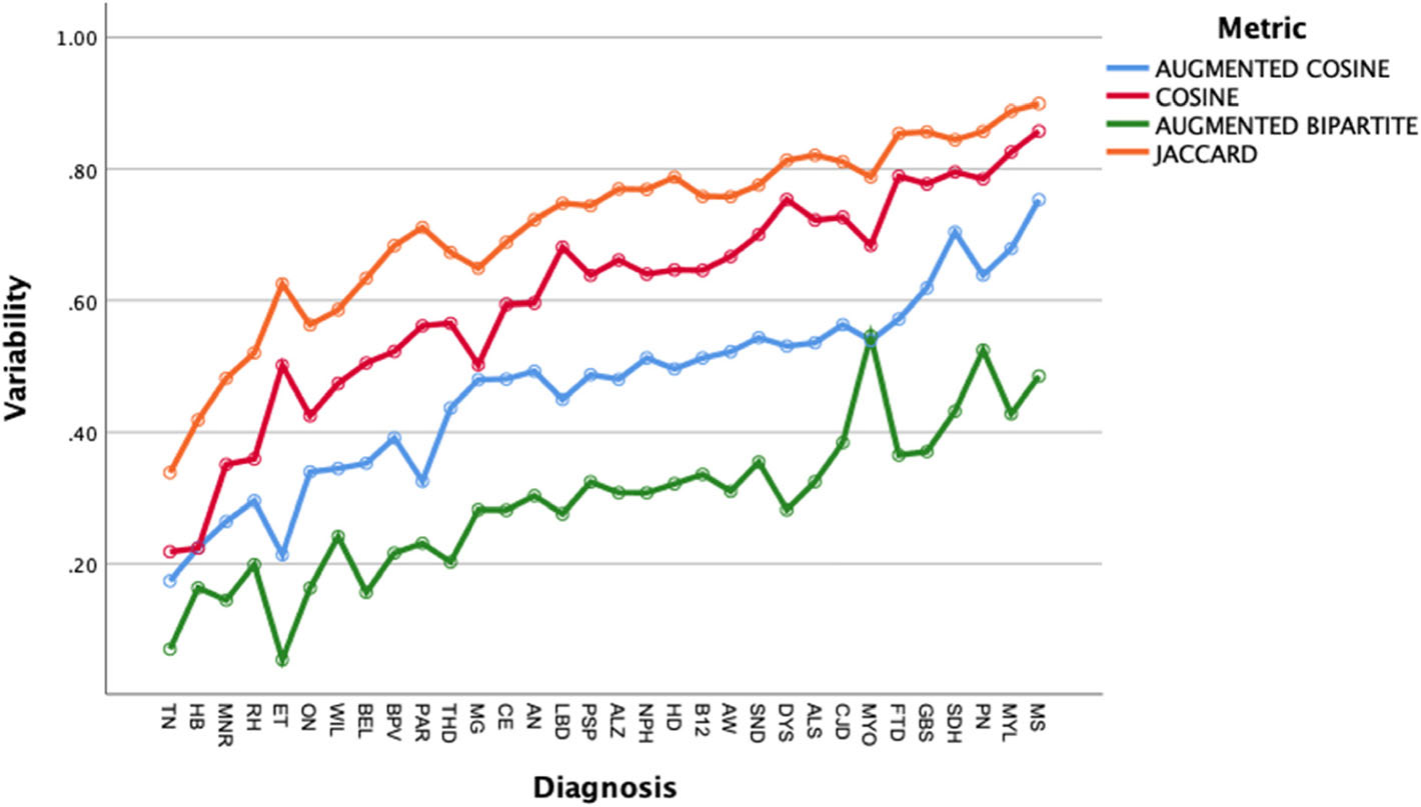
Mean within diagnosis distance by diagnosis in ascending order. Greater within-diagnosis mean patient distance suggests greater variability of clinical presentation within a diagnosis. Diagnoses that are most variable in clinical presentation are to the right of the x-axis. Within-diagnosis mean patient distances vary by diagnosis (two-way ANOVA, df = 31, p < .05)

We found a significant difference in mean patient distances by diagnosis (Fig. 3, two-way ANOVA, means differ by diagnosis, df = 31, F = 107, p < .001, and means differ by distance metric, df = 3, F = 1351, p < .001). Post hoc Bonferroni testing showed that 60% of the pairwise patient distance means differed by diagnosis (*P* < .05). For the 32 diagnoses shown in Fig. 3, trigeminal neuralgia has the lowest mean within-diagnosis patient distance (less than all other 31 diagnoses, pairwise comparisons, p < .05) and multiple sclerosis had the highest within-diagnosis mean patient distance (greater than all other diagnoses, pairwise comparisons, p < .05).

We performed 64 classification analyses (4 distance metrics × 4 test groups × 4 classifiers). The four test groups were *altered mental status*, *abnormal movement, cranial neuropathy, and weakness* (Table 2). The four distance metrics were cosine, augmented cosine, augmented bipartite, and Jaccard (see Methods). The four classifiers were naïve Bayes, logistic regression, random forest, and k-nearest neighbor (k = 5). Classes were unbalanced in the test groups (Table 2). Each classification task involved selecting the correct diagnosis from one of eight competing diagnoses for each of the patients in the test group. The performance was measured by classification accuracy and F1. Classification performance varied by classifier for both classification accuracy (two-way ANOVA, main effect, df = 3, F = 7.8, *p* < .001) and F1 (two-way ANOVA, main effect, dF = 3, F = 10.1, p < .001). Bonferroni post hoc testing showed that the naïve Bayes classifier underperformed the logistic regression and k-nearest neighbor classifiers on both performance measures (*p* < .05).

**Fig. 4:**
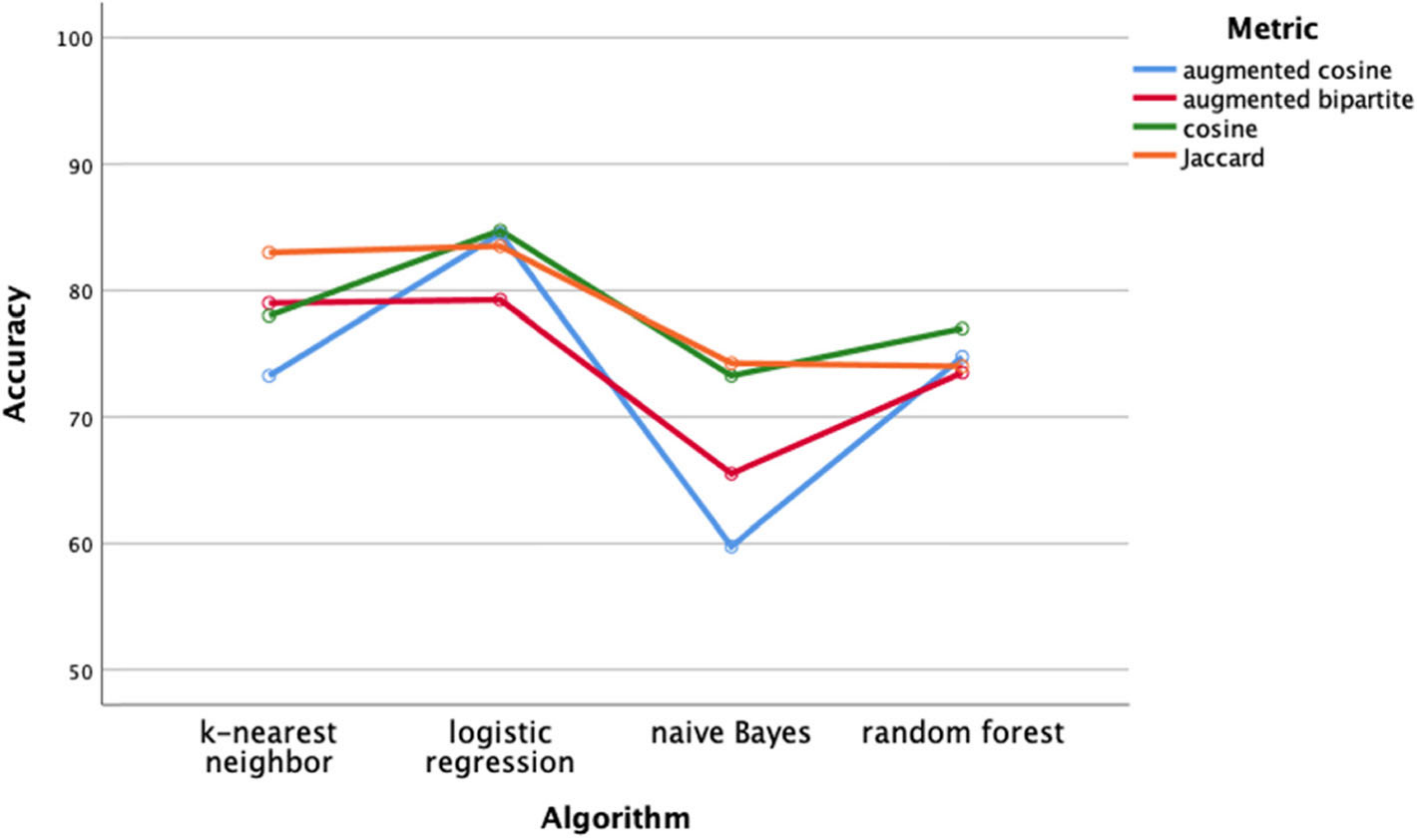
Performance of classifiers by distance metric assessed by classification accuracy. Classification performance on classifiers did not vary by distance metric (*p* > .05). The k-nearest neighbor and logistic regression classifiers outperformed the naïve Bayes classifier (Bonferroni post hoc test, p < .05)

**Fig. 5:**
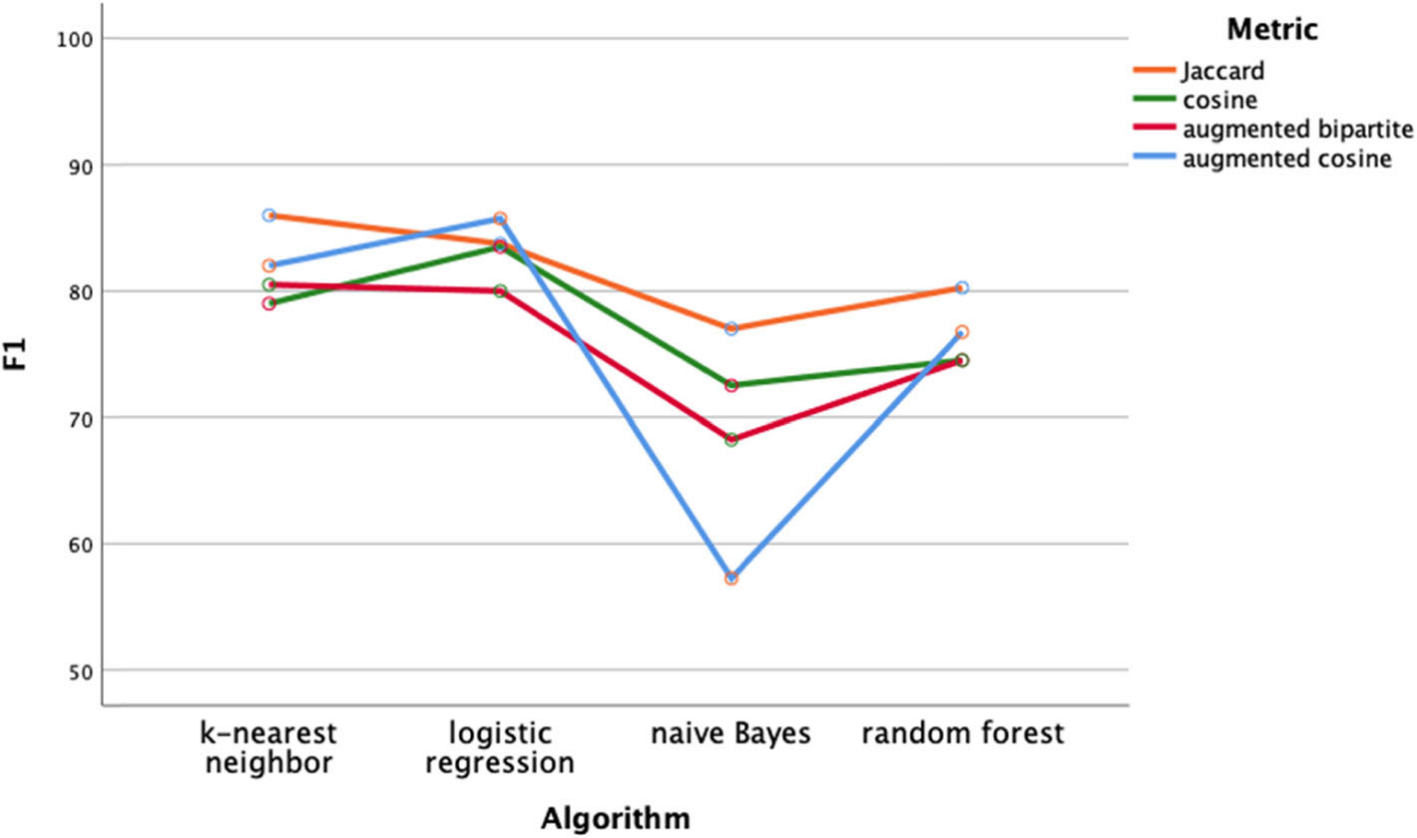
Performance of classifiers by distance metric assessed by balanced F1. Balanced F1 did not vary by distance metric (two-way ANOVA, df = 3, p > .05). Naïve Bayes underperformed the k-nearest neighbor and logistic regression classifiers (p < .05)

**Fig. 6:**
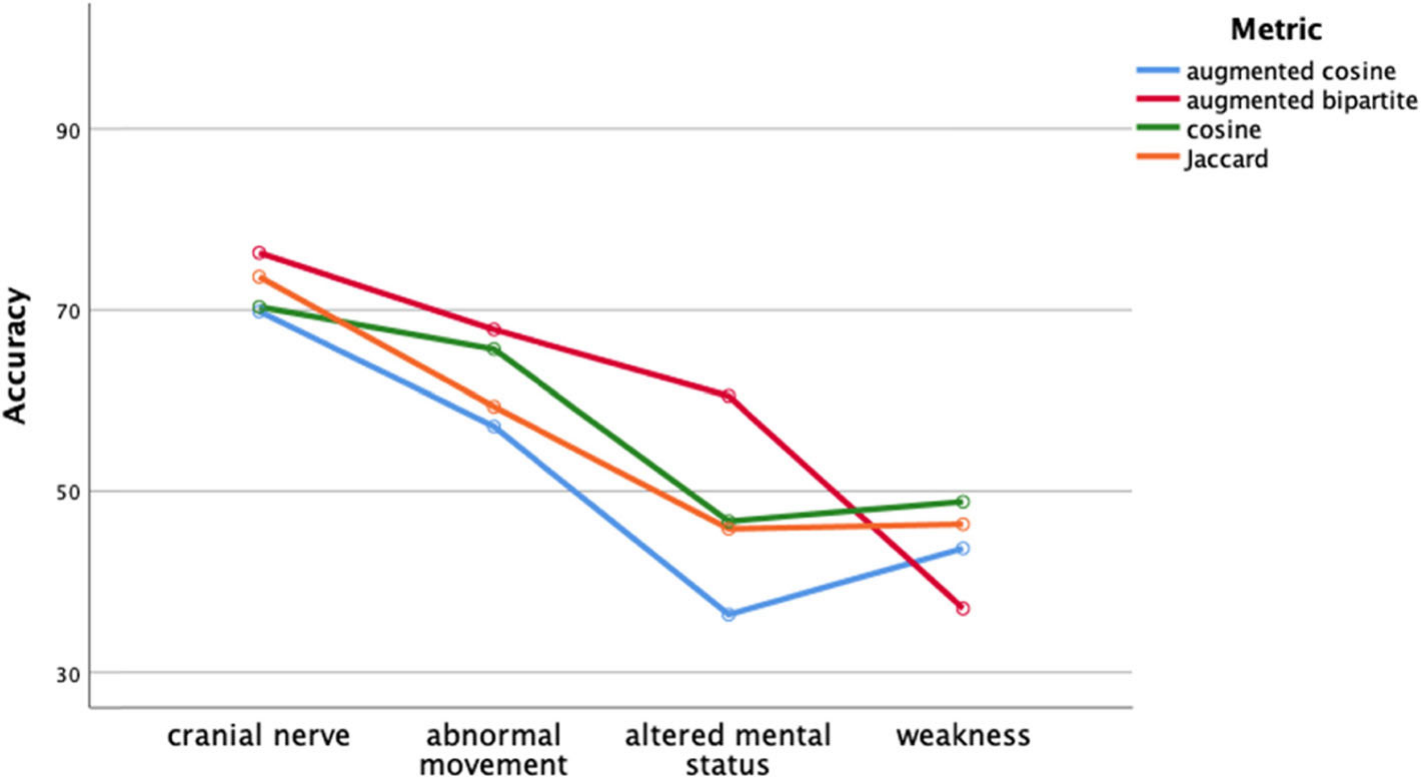
Mean performance of all classifiers by test group assessed by classification accuracy. Classification accuracy did not vary by distance metric (two-way ANOVA, df = 3, p > .05). Classification accuracy was higher for the cranial nerve group than the other diagnosis groups (Two-way ANOVA, df = 3, *p* < .01, post hoc Bonferroni test, p < .05)

**Fig. 7:**
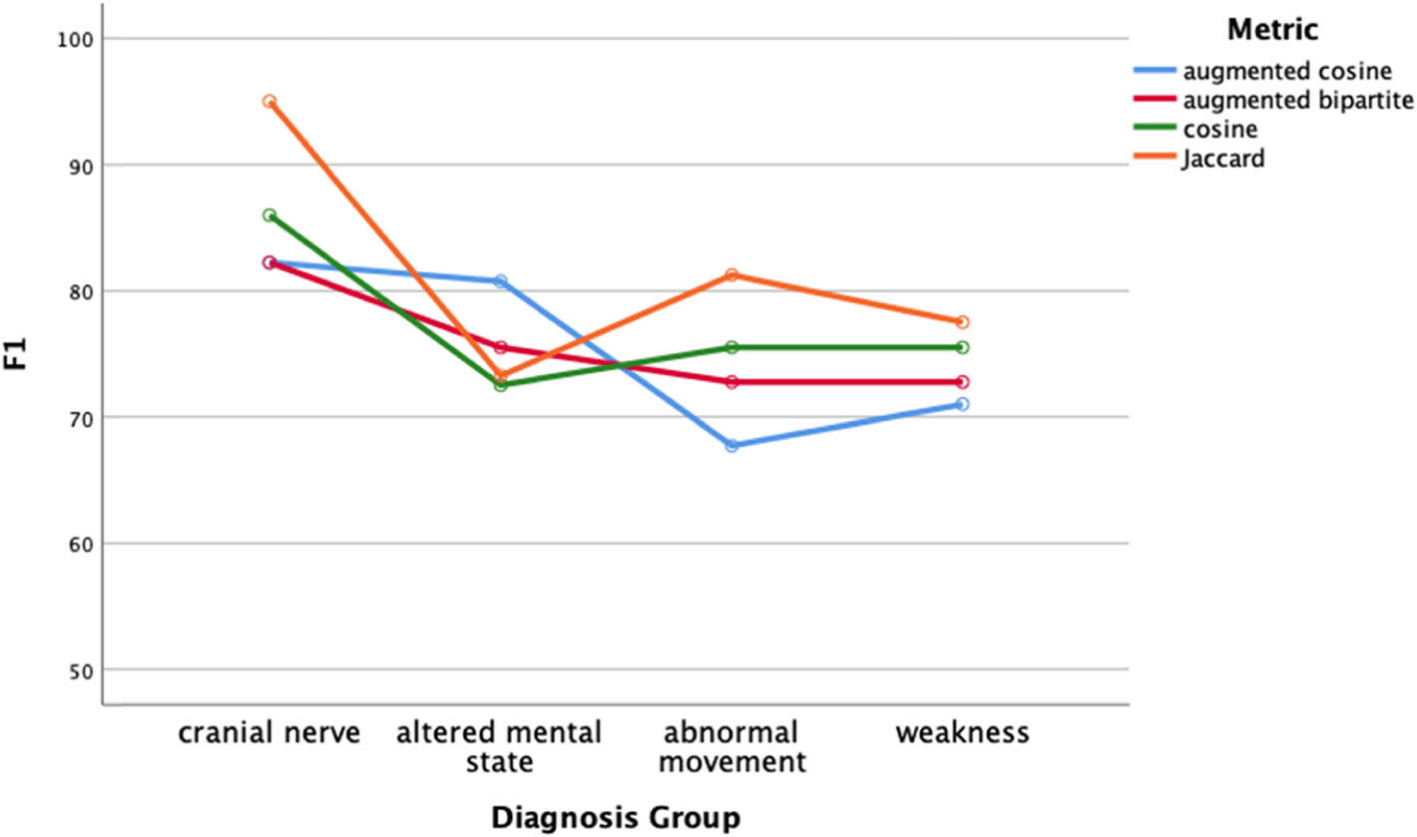
Mean performance of all classifiers assessed by F1 by test group and distance metric. F1 did not vary by distance metric (Two-way ANOVA, df = 3, p > .05). F1 varied significantly by diagnosis group (df = 3, p < .001, F1 was higher for the cranial nerve test group, p < .05, post hoc Bonferroni test)

Classification performance of the distance metrics was comparable regardless of classifier (Figs. 4-5, two-way ANOVA, df = 3, *p* > .05) or diagnosis group (two-way ANOVA, Figs. 6-7, df = 3, p > .05). Classifier performance was comparable when performance was measured by classification accuracy (Figs. 4) or by F1 (Fig. 5). Performance differed by diagnosis group (Figs. 6 and 7) for both classification accuracy (two-way ANOVA, df = 3, F = 10.2, p < .001) and the F1 score (two-way ANOVA, df = 3, F = 7.4, *P* < .001). Post hoc Bonferroni testing showed the classification accuracy score, and the F1 score was higher for the cranial nerve group than the other three diagnosis groups (*p* < .05).

**Fig. 8:**
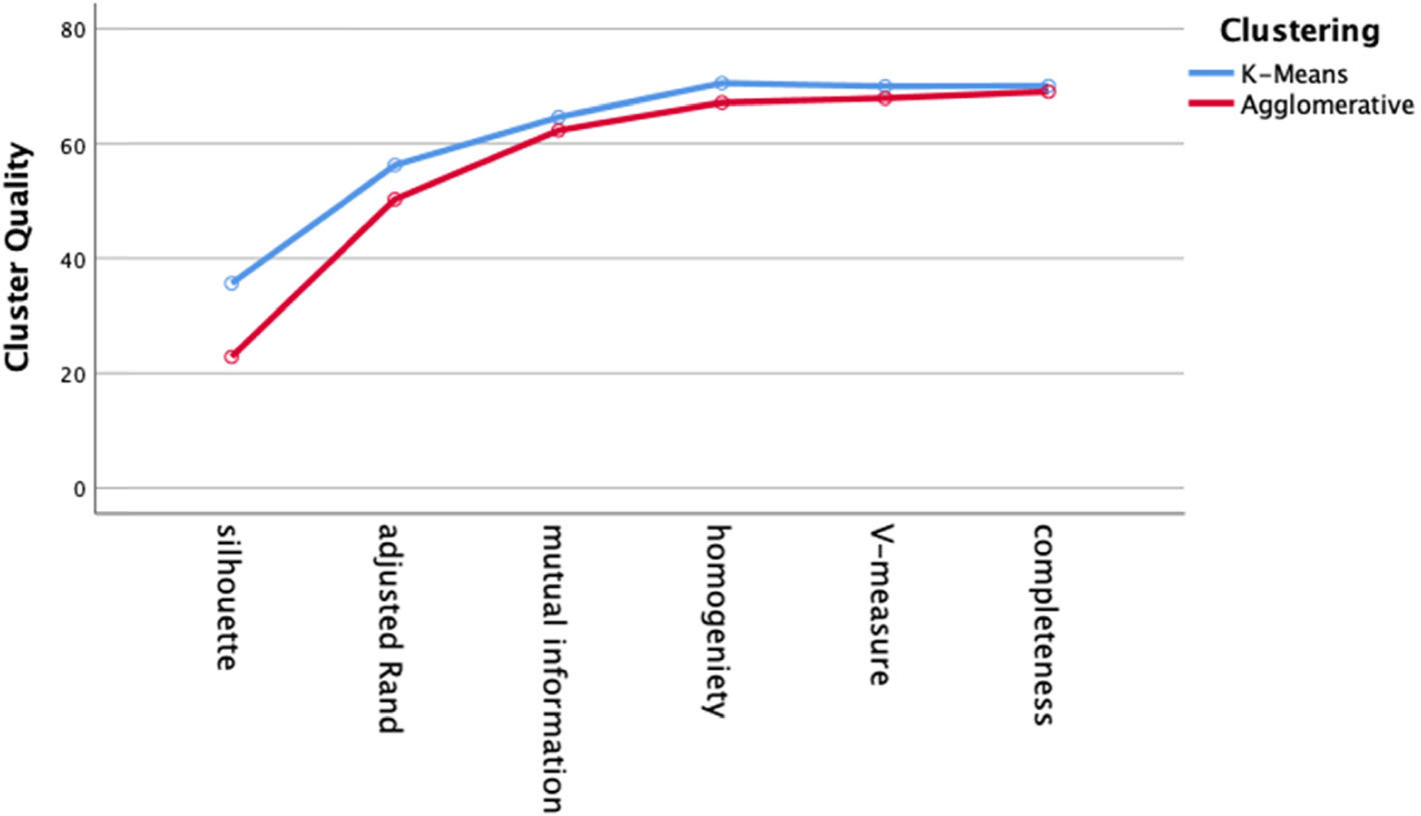
Cluster quality for all test groups comparing k-means to agglomerative clustering (all distance metrics). Cluster quality did not differ by clustering algorithm (two-way ANOVA, df = 1, p > .05)

We performed 32 clustering analyses (4 distance metrics × 4 test groups × 2 clustering algorithms). The two clustering algorithms were agglomerative clustering with Ward linkage and k-means clustering. Distances were inputted as pre-computed *nxn* matrices. For both clustering algorithms, the number of clusters was set at eight based on the known number of different diagnoses in each diagnosis group. Cluster quality was assessed by silhouette score, adjusted Rand Index (ARI), adjusted mutual information (AMI), completeness, homogeneity, and V-measure. Cluster quality did not differ by cluster algorithm (agglomerative versus k-means) on any of the cluster quality measures (Fig. 8, two-way ANOVA, df = 1, *p* > .05).

**Fig. 9:**
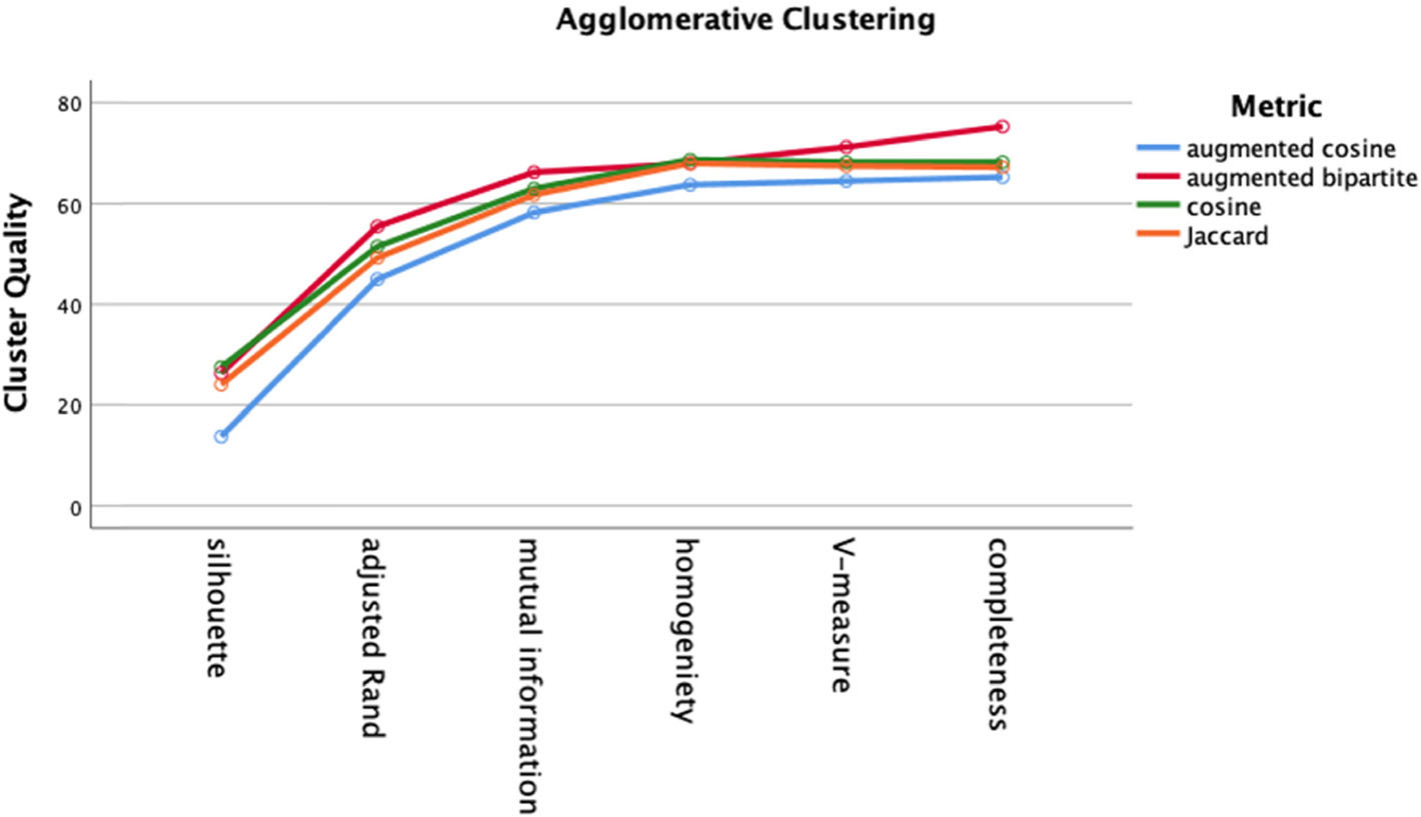
Cluster quality for agglomerative clustering by distance metric. Cluster quality did not differ by distance metric (two-way ANOVA, df = 3, p > .05)

**Fig. 10:**
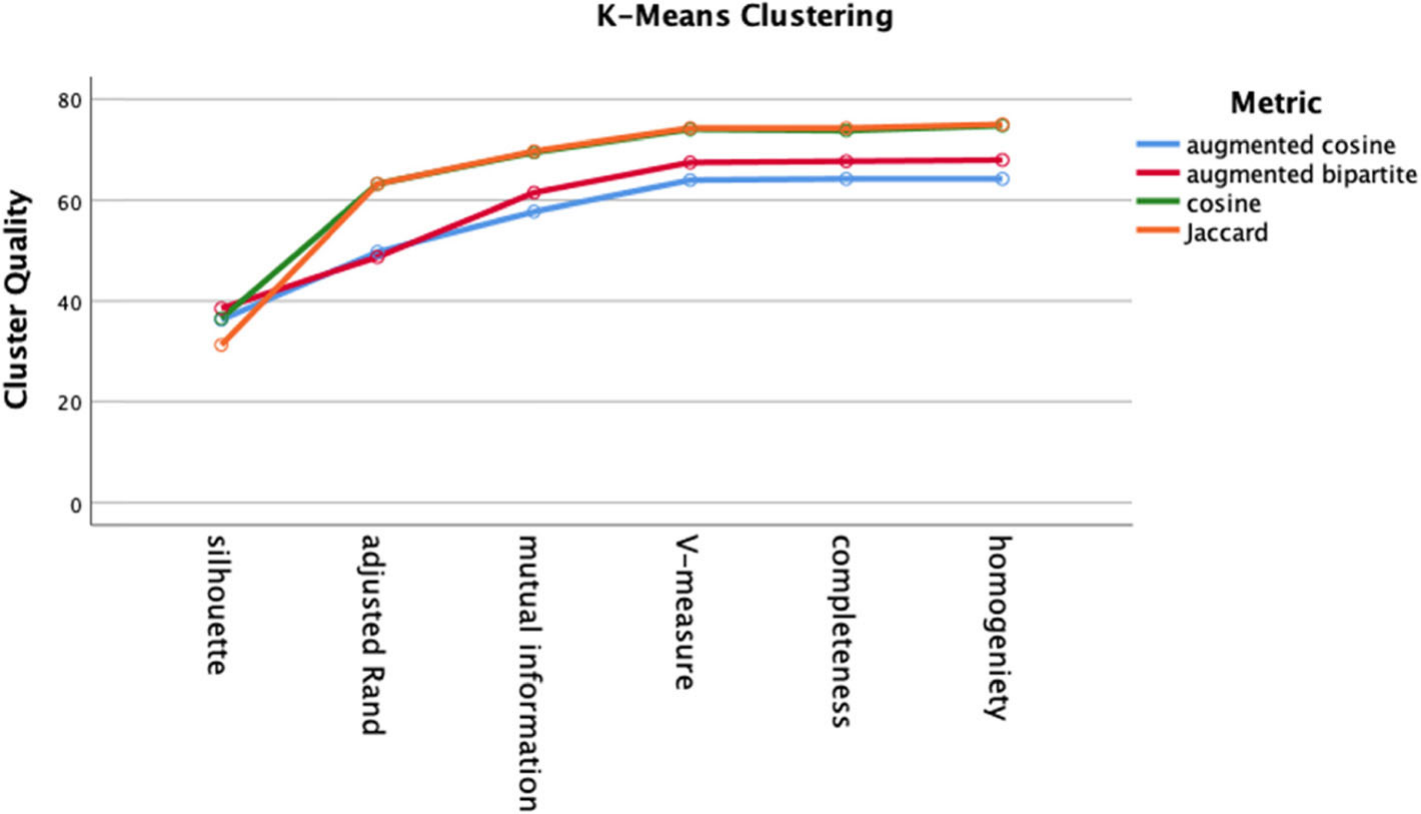
Cluster quality of k-means clustering by distance metric. Cluster quality did not differ by distance metric (two-way ANOVA, df = 3, p > .05)

**Fig. 11:**
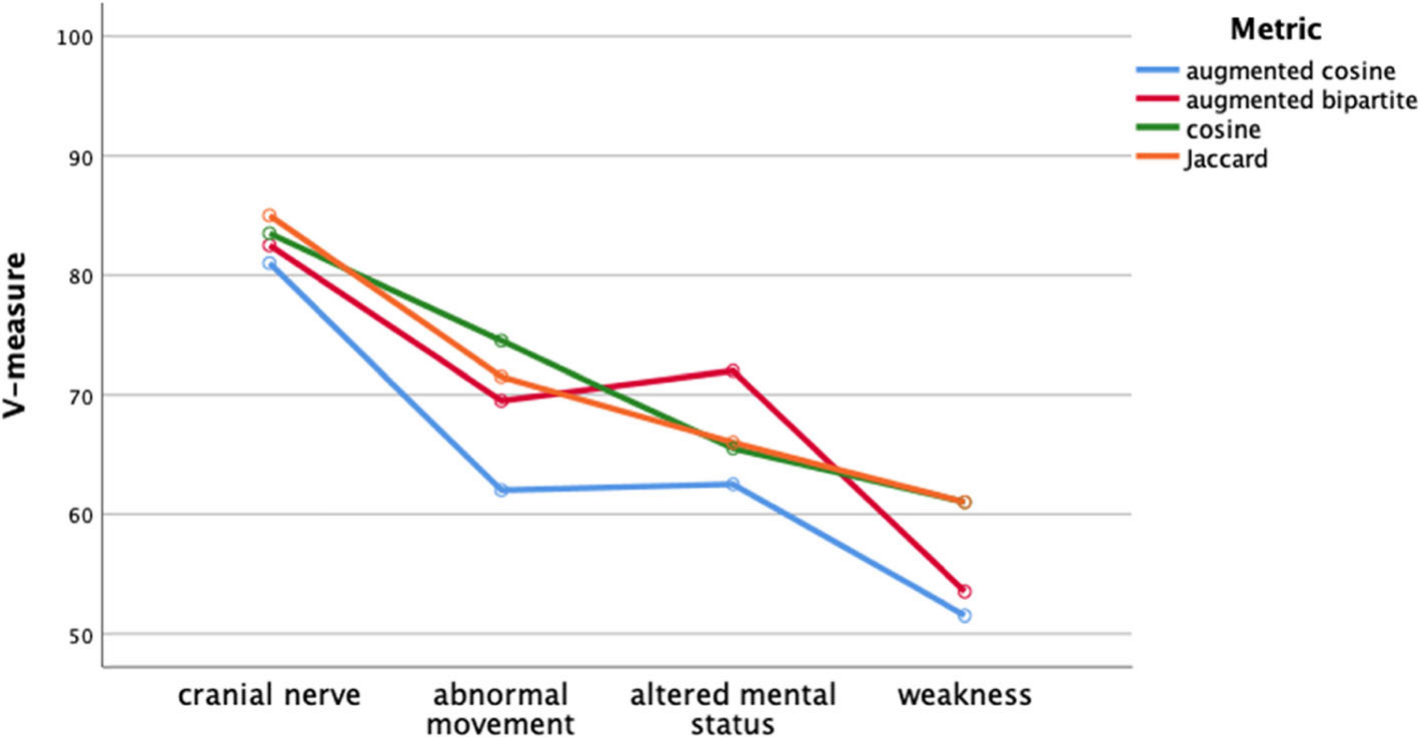
Cluster quality assessed by V-measure by test group and distance metric. Cluster quality did not vary by distance metric (df = 3, p > .05). V-measures varied by diagnosis group (two-way ANOVA, df = 3, p < .001; post hoc Bonferroni testing showed cranial nerve group to have higher cluster quality, p < .05)

**Fig. 12:**
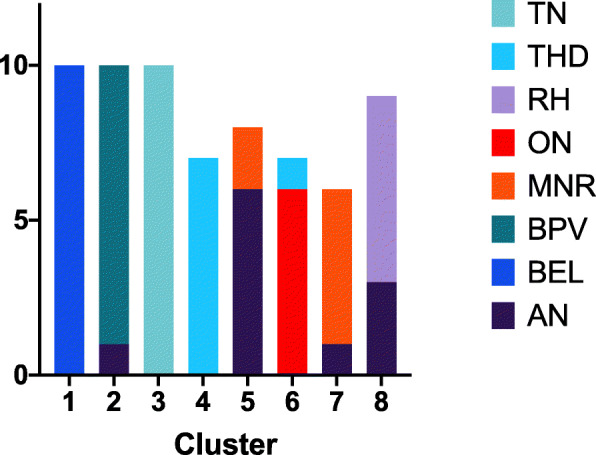
Distribution of ground truth diagnoses by cluster for the cranial nerve test group. K-means clustering with Jaccard distance metric. Each color represents a different ground truth diagnosis. Each column represents a different computed cluster. Homogeneity for the cranial nerve group is greater than for the weakness group (see Fig. 13)

**Fig. 13:**
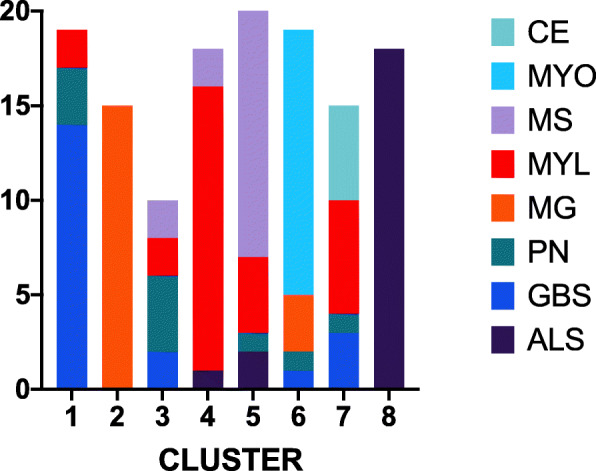
Distribution of ground truth diagnoses by cluster for the weakness test group. K-means clustering with Jaccard distance metric. Each color represents a different ground truth diagnosis. Each column represents a different computed cluster. Homogeneity for the weakness group is less than for the cranial nerve group. (see Fig. 12)

For both k-means clustering and agglomerative clustering, the distance metric did not significantly affect cluster quality (Figs. 9 and 10, two-way ANOVA, df = 3, p > .05). Cluster quality was better for the cranial nerve group (Fig. 11) than the other three groups, the movement group was better than the weakness group (Bonferroni post hoc test, p < .05; Groups differ two-way ANOVA, df = 3, F = 20.3, *p* < .001). The higher quality of the cranial nerve clustering with greater within-cluster homogeneity than the weakness group clustering is illustrated in the stacked bar charts Figs. 12 and 13.

## Discussion

We examined four distance metrics for calculation of the distances between neurology patients based on findings: Jaccard distance, cosine distance, augmented cosine distance and augmented bipartite distance. To calculate the Jaccard and augmented bipartite distances, we represented patients as unordered lists of elements of variable length (sets). To calculate the cosine and augmented cosine distances, we represented patients as ordered arrays of fixed length (vectors).

For the Jaccard and cosine distances, the matching of concepts between patients was binary (“all or none”). Semantic similarity between concepts was not considered. Consider a patient A that has the finding *resting tremor*; and a patient B that has the finding *postural tremor*. When calculating the Jaccard distance or the cosine distance, the semantic similarity between *resting tremor* and postural tremor would not contribute to the proximity between these two patients (each metric would value the similarity between *resting tremor* and *postural tremor* as ‘0’). The semantically augmented distance metrics behave differently. These augmented distance metrics move patients closer together when patients manifest semantically similar findings, even if they are not exact matches. The augmented cosine distance considers that postural tremor and *resting tremor* have a common immediate ancestor *tremor*. Hence, the *tremor* element of the vectors for patient A and patient B is augmented with a value of 0.5 (see Methods and [19]). This semantic augmentation of the vectors for patients A and B increases their similarity and moves the patients closer together when the cosine distance is calculated (eq. 8). The augmented bipartite distance considers that *resting tremor* and *postural tremor* are siblings in the neuro-ontology hierarchy and have a Wu Palmer distance of 0.25 (eq. 7); moving patients A and B closer (eqs. 5 and 6). The augmented cosine distance metric moves the patients closer because *postural tremor* and *resting tremor* have *tremor* as a common ancestor in the neuro-ontology. The augmented bipartite distance metric moves the patients closer because *resting tremor* and *postural tremor* are siblings in the neuro-ontology.

For each of the 382 patients in the dataset (*n* = 382), we calculated the mean patient distance to patients with the same diagnosis and the mean distance to patients with different diagnoses (Fig. 2). Within-diagnosis patient distances were lower than between-diagnosis patient distances for all of the metrics (Fig. 2). Patients of the same diagnosis should be closer to each other than those with a different diagnosis. Sematic augmentation of the distance metrics makes patients more similar, moves them closer together, and reduces mean patient distances. Augmented cosine and augmented bipartite patient distances were lower than cosine and Jaccard patient distances (Fig. 1, Bonferroni post hoc test, *p* < .05). For each patient, the difference between its mean distance to other patients with the same diagnosis and its mean distance to other patients with different diagnosis (Fig. 2) is important because it is this difference between within-diagnosis and between-diagnosis distances that contributes to the ability of clustering and classification algorithms to use distances to cluster or classify patients by patient distance successfully [63, 64]. The difference between mean within-diagnosis distance and mean-between diagnosis distance differed by metric (df = 3, F = 49, *p* < .001) with the largest differences found with the cosine and augmented cosine metrics and the smaller differences found with the augmented bipartite and Jaccard metrics (Bonferroni post hoc test, p < .05).

### Classification and clustering

We evaluated four different classifiers on four different test groups of patients. We used F1 and classification accuracy (Figs. 4 and 5) as measures of classification performance. There were differences in classifier performance, with the logistic regression classifier and the k-nearest neighbor classifier outperforming the naïve Bayes classifier (Figs. 4 and 5). In retrospect, the selection of the naïve Bayes classifier was ill-suited for this study since this classifier assumes feature independence (not likely to hold among neurological patients) and is oriented towards using probabilities rather than distances for classification. Importantly, we found no effect on classification performance related to the distance metric. Classification performance did vary by test group (Figs. 6 and 7). Post hoc testing showed that the classification performance was better for the cranial nerve test group. A likely explanation for the better classification performance with the cranial nerve group is that members of this group (Table 2) had tighter within diagnosis inter-patient distances (i.e., less variability in presentation). As illustrated in Fig. 3, the diagnoses of the cranial nerve test group (TN, MNR, RH, ON, BEL, BPV, THD, and AN) are primarily on the left-hand side of the x-axis, and they have lower mean intra-diagnosis variability in their clinical presentations.

We evaluated two different clustering algorithms (agglomerative clustering and k-means clustering) on the four test groups of patients (Table 2). Except for the silhouette score, the clustering performance measures depend on the ground truth diagnosis label derived from the patient case studies. The silhouette score measures cluster quality independent of ground truth. Cluster quality did not differ by cluster algorithm (Fig. 8). Cluster quality did not vary by distance metric for either the k-means algorithm or the agglomerative algorithm (Figs. 9 and 10). Cluster quality did differ by patient test group with post hoc testing showing that the cranial nerve test group had higher cluster quality than the other test groups (Fig. 11). Visual inspection of Figs. 12 (cranial nerve test group) and Fig. 13 (weakness test group) show how with an 8-cluster solution, cluster *homogeneity* is higher in the cranial nerve group than the weakness test group. In Figs. 12 and 13, each color represents a different ground truth diagnosis label, and each column represents a computed cluster. The better performance on clustering of the cranial nerve group likely reflects the same factors intrinsic to this group of patients that led to better classification performance (see above). There is less variability in clinical presentation from patient to patient in this test group, within-diagnosis patient distances are lower (Fig. 3), and there is likely less sign and symptom overlap with other diagnoses.

The failure to find an improvement in clustering or classification performance with semantically augmented distance measures was somewhat surprising. Others have found improvements in the clustering of patients [13] or classification of documents [19] with semantically augmented distance metrics. However, Melton et al. [16] did not find improved concordance with domain experts when inter-patient distance calculations were augmented with concept semantic similarity information. Although semantically augmented distance metrics move patients closer (Fig. 1), these smaller inter-patient distances may not translate into improvements in clustering or classification performance unless these smaller distances create a greater gap between mean within-diagnosis distance and mean between-diagnosis. From Fig. 2, it seems likely that for patients with a given diagnosis, semantic augmented distance places them closer to other patients with the same diagnosis. The problem is that semantically augmented distances push these patients closer to other patients with a different diagnosis. If the net effect of semantic augmentation is to make each patient closer to patients with the same diagnosis and patients with a different diagnosis, there will be no net gain in the ability to cluster or classify patients by diagnosis. The non-intuitive failure of semantic augmentation to improve classification and clustering performance can be illustrated by returning to the hypothetical patient A with *resting tremor* and the hypothetical patient B with *postural tremor.* If the diagnosis of patient A is Parkinson disease and the diagnosis of patient B is essential tremor (as is likely), then semantically augmented distance metrics will move patient A closer to B. However, since the diagnosis of patient A and patient B are different, moving patient A closer to patient B will deprecate classification and clustering performance in this case.

### Implications for neurological diagnosis

The accuracy of diagnosis for the 32 neurological diagnoses in this study ranged from 76 to 86% with the k-nearest neighbor classifier (Fig. 4). In one study, human experts made neurologic diagnoses at the bedside with an accuracy of 77% [65]. Liu et al. [66] observe “machine learning methods can only be as good as the information in the training set … machine-learning methods should not be able to exceed the performance of extremely careful and experienced clinicians …. ” Machine learning can offer insights into which diseases are more variable in presentation than others (Fig. 3) and which diagnostic problems are more challenging to solve than others (Fig. 6). Furthermore, machine learning may offer improvements in patient matching strategies for large repositories of archetypal disease profiles such as the Online Mendelian Inheritance in Man [4, 5, 12].

### Limitations

One limitation of this study is that we did not consider the severity of deficits, such as weakness or ataxia. When deficits were present, they were binarized as either present or absent and not graded in severity. Another limitation is that some of the diagnosis classes were narrower than others. Although some of the diagnosis classes were specific (Huntington disease, Alzheimer disease, and Parkinson disease), others were more general, such as polyneuropathy, myopathy, and meningitis. This decision to use more general categories for some diagnosis classes reflects the reality that signs and symptoms alone are unlikely to distinguish specific causes of meningitis, polyneuropathy, or myopathy without additional ancillary testing. Another limitation is that we did not compare the computed patient distances to expert opinion for any of the distance metrics. The validity of the results would be improved by a larger dataset of patients, preferably in the thousands rather than in the hundreds. A further limitation of the study is that we utilized published cases from the textbooks of neurology rather than de-identified patient records from electronic medical records. We used manual abstraction of concepts from case histories instead of natural language processing (NLP) [67–70]. We chose manual abstraction rather than NLP because we wanted to carefully curate a database of test patients with minimal coding errors, and our initial experience with MetaMap indicated that extensive post-processing was needed to ensure accuracy. Future advances in NLP could make the conversion of signs and symptoms in electronic health records to machine-readable codes more accurate and efficient. Inter-rater reliability for abstracting clinical cases into UMLS codes or SNOMED CT codes is another concern [20, 21].

## Conclusions

Neurological signs and symptoms from case histories can be represented as UMLS concepts from a neuro-ontology. We examined four different distance metrics for the calculation of inter-patient distances. All of the distance metrics provided useful patient distances that could be utilized by machine learning classification and clustering algorithms. Semantically augmented metrics that used the semantic similarity between neurological concepts to calculate patient distances yielded lower patient distances than more traditional distance metrics without semantic augmentation. When each of the four distance metrics was tested on four classifiers and two clustering algorithms, all distance metrics performed similarly without a discernible improvement due to semantic augmentation. Further work is needed to determine the utility of semantically augmenting patient distance metrics with inter-concept distances.

## Data Availability

Neurology cases are available at 10.17632/z3d6hwrdmh.2 Inter-concept distances are available at 10.17632/svrx3wgcn4.3 Inter-patient distances are available at 10.17632/svrx3wgcn4.4

## Abbreviations

CUI: UMLS concept unique identifier
UMLS: Unified Medical Language System
SNOMED CT: is a registered name of SNOMED International.
NLP: Natural language processing
HPO: Human Phenotype Ontology
OMIM: Online Mendelian Inheritance in Man
